# Rational Medicine—A Review*Brief Expositions of Rational Medicine, to which is prefixed the Paradise of Doctors, a fable. By Jacob Bigelow, M. D., late President of the Massachusetts Medical Society, Physician of the Massachusetts General Hospital, &c. Boston: Phillips, Sampson & Co., 13 Winter St. 1858. 12 mo. pp. 69.

**Published:** 1858-11

**Authors:** 


*Rational Medicine—A Review.** Communicated for the Boston Medical and Surgical Journal.

The Profession and the Public are under renewed obligations to Dr. Bigelow for the publication of this little volume. In it he advocates afresh those principles on which just ideas of the true mission and powers of the medical art must be forever founded, and vindicates a doctrine to which its followers in this vicinity have for some time past given the title of "Rational Medicine," hoping themselves to merit in some reasonable measure the distinctive appellation of "Rational Physicians," in contradistinction to such as may pursue an artificial, heroic, expectant, homœopathic or any more narrow system of practice.

An early promoter of these principles, and for a quarter of a century their especial advocate, Dr. Bigelow, by various publications, and still more by the unmeasured influence of learning and experience, combined with strong common-sense philosophy, has held a salutary check upon medical assumption and credulity, and effected a marked change in the reasonings and general practice of physicians in this part of the country. It is not too much to say that to him, more than all others, is due the commanding respect which "Rational Medicine" is now receiving and is henceforth to receive on this side of the Atlantic, over the "extravagances of a so-called heroic and over-active practice on the one hand, and of a nugatory and ignorant practice on the other."

The publication before us seems to have been written as an introduction to a series of re-publications which Dr. Bigelow had proposed to issue under the general title of "Rational Medicine," but in which he was interrupted by the unforseen appearance of a New York edition of Sir John Forbes' "Nature and Art in the cure of Disease," which was to have formed the first volume of the series. It is therefore naturally dedicated to that gentleman, with a just tribute to the great service he has rendered the cause in former times, and more especial-

*Brief Expositions of Rational Medicine, to which is prefixed the Paradise of Doctors, a fable. By Jacob Bigelow, M. D., late President of the Massachusetts Medical Society, Physician of the Massachusetts General Hospital, &c. Boston: Phillips, Sampson & Co., 13 Winter St. 1858. 12 mo. pp. 69.

ly by his recent " noble work " connected with the subject above named. After the dedication, follows an allegory, read at the Annual Dinner of the Massachusetts Medical Society, entitled the " Paradise of Doctors," in which the past and present condition of medical matters is set forth in an entertaining manner, and with more truth, we fear, than fable.

In coming to the body of the publication, from which it was the purpose of this article to make a few quotations, we find it almost impossible to make the selection, so important and truthful is every word and sentence throughout its invaluable pages.

" The methods which, at the present day, are most prevalent in civilized countries, in the treatment of disease, may be denominated the following :

"2. The *Artificial* method, which, when carried to excess, is commonly termed heroic, and which consists in reliance on artificial remedies, usually of an active character, in the expectation that they will of themselves, remove diseases.

" 2. The *Expectant* method. This consists simply in non-interference, leaving the chance of recovery to the powers of nature, uninfluenced by interpositions of art.

" 3. The *Homœopathic* method. This is a counterfeit of the last, and consists in leaving the case to nature, while the patient is amused with nominal and nugatory remedies.

" 4th. The *Exclusive* method, which applies one remedy to all diseases or a majority of diseases. This head includes hydropathy, also the use of various mineral waters, electrical establishments, etc. Drugs newly introduced, and especially secret medicines, frequently boast this universality of application.

" 5. The *Rational* method. This recognizes nature as the great agent in the cure of diseases, and employs art as an auxiliary, to be resorted to when useful or necessary, and avoided when prejudicial.

" The foregoing methods, with the exception perhaps of the last, have had their trial in various periods and countries, and have given rise to discussions and controversies which are not terminated at the present day.****Any person who will take the trouble to inspect the medical journals published thirty or forty years ago will find many things, then laid down as medical truths, which are now generally admitted to be medical errors." Pp. 27-28.

" The vulgar estimate of the power of medicine is founded on the common acceptance of the name, that medicine is the art of curing diseases. A far more just definition would be, that medicine is the art of understanding diseases, and of curing or relieving them when possible. If this definition were accepted, and its truth generally understood by the profession and the public, a weight of superfluous responsibility on one side, and of dissatisfaction on the other, would be lifted from the shoulders of both. It is because physicians allow themselves to profess and vaunt more power over disease than belongs to them, that their occasional short comings are made a ground of reproach with the community, and of contention among themselves.

"It is now generally admitted by intelligent physicians that certain diseases, the number of which is not very great, are at once curable by medical means. It is also beginning to be admitted in this country that certain diseases are *self-limited*,—incurable now by art, yet susceptible of recovery under natural processes, both with and without the interference of art. Yet, so reluctant are physicians to acknowledge these universal truths or to admit their own competency, that incurable and unmanageable diseases have been complacently call *opprobria medicinæ*, as if they were exceptions to a general rule." Pp. 29–31.

"The safe conduct of the sick, as will be seen from the last head, consists much more in cautionary guidance than in active interference. ****People sometimes suffer from neglect, but more frequently from ill-judged and meddlesome attention.****Intelligent and discreet physicians are sometimes driven by the importunity of friends to the adoption of active measures, or at least the semblance of them, which their own judgement informs them would be better omitted. And the case is still worse when the impulsive temperament of the physician himself, or the influence of his early education, or the dominant fashion of the place in which he resides, is so exacting in regard to the activity of treatment as to make him believe that he cannot *commit* too many inflictions upon the sick, provided that, in the end, he shall be satisfied that he has *omitted* nothing." Pp. 33–34.

"From the earliest ages a belief has prevailed that all human maladies are amenable to contrel from some form of purely medical treatment and although the precise form has not been found, so far as most diseases are concerned, yet, at this day, it continues to be as laboriously and hopefully pursued, as was the elixir vitæ in the middle ages. Within the present century, books of practice gravely laid down "the indications of cure" as if they were things within the grasp of every practitioner. It was only necessary to subdue the inflammation, to expel the morbific matter, to regulate the secretions, to improve the nutrition, and to restore the strength, and the business was at once accomplished. What nature refused, or was inadequate to do, was expected to be achieved by the more prompt and vigorous interposition of art. The destructive tendencies of disease, and the supposed proneness to deterioration of nature herself, were opposed by copious and exhausting depletion, followed by the shadowy array of alteratives, deobstruents and tonics. Confinement by disease, which might have terminated in a few days, was protracted to weeks and months, because the importance of the case, as it was thought, required that the patient should be artificially 'taken down,' and then artificially 'built up.'

"When carried to its 'heroic' extent, artificial medicine undermined the strength, elicited new morbid manifestations, and left more disease than it took away.****A considerable amount of violent practice is still maintained by routine physicians, who, without going deeply into the true nature or exigencies of the case before them, assume the general ground that nothing is dangerous but neglect.****Consulting physicians frequently and painfully witness the gratuitous suffering, continued nausea, the prostration of strength, the prevention of appetite, the stupefaction of the senses, and the wearisome days and nights, which never would have occurred had there been no such thing as officious medication.****If diseases proved fatal, or even if they were not jugulated or cut short at the outset, the misfortune was attributed to to the circumstance of the remedies not being sufficiently active, or of the physician not being called in season. So great at one time, and that not very long ago, was the ascendency of heroic teachers and writers, that few medical men had the courage to incur the responsibility of omitting the active modes of treatment which were deemed indispensable to the safety of the patient." Pp. 35-38.

"I sincerely believe that the unbiassed opinion of most medical men of sound judgment and long experience is made up, that the amount of death and disaster in the world would be less, if all disease were left to itself, than it now is under the multiform reckless and contradictory modes of practice, good and bad, with which practitioners of adverse denominations carry on their differences at the expense of their patients." Pp. 41.

"It is to sincere and intelligent observers, and not to audacious charlatans, that we are to look as the ultimate law-givers of medical science. Our present defect is not that we know too little, but that we profess too much. We regard it as a sort of humiliation to acknowledge that we cannot always cure diseases, forgetting that in many other sciences mankind have made no greater advances than ourselves, and are still upon the threshold of their respective structures. Medical assumption may well feel humbled by the most insignificant diseases of the human body. Take, for example, a common furunculus or boil. No physician can, by any internal treatment, produce it where it does not exist. No physician can, by any science, explain it, and say why it came on one limb and not npon another. No physician can, by any art, cure it, after it has arrived at a certain height. No physician can by any art, delay or retain it after it has passed the climax assigned to it by nature. And what is true in regard to a boil, is equally true of common pneumonia, of typhoid fever, of acuterheu matism, of cholera, and many other diseases.

"In the present state of our knowledge the truth appears to be simply this: Certain diseases, of which the number is not very great, are curable or have their cure promoted, by drugs, and by appliances which are strictly medicinal. Certain other diseases, perhaps more numerous, are curable in like manner by means which are strictly regimenal, and consist in changes of place, occupation, diet, and habits of life. Another class of diseases are self-limited, and can neither be expelled from the body by artificial means, nor retained in the body after their natural period of duration has expired. Finally, a large class of diseases have proved incurable from the beginning of history to the present time, and under some one of these the most favored member of the human race must finally succumb; for even curable diseases become incurable when they have reached a certain stage, extent, or complication.****

"It is the part of rational medicine to study intelligently the nature, degree and tendency, of each existing case, and afterwards to act, or to forbear acting, as the exigencies of such case may require. To do all this wisely and efficiently, the practitioner must possess two things: first, to diagnosticate the disease; and, secondly, sufficient sense to make up a correct judgment on the course to be pursued." Pp. 48–50.

"Having already touched upon this subject, I have only to add, that if many of the troublesome appliances and severe exactions of modern practice were superseded by gentler, more soothing, and more natural means, a good wonld be done to the human race comparable to the conversion of swords into ploughshares." Pp. 54.

"It is the part of rational medicine to require evidence for what it admits and believes. The cumbrous fabric now called therapeutic science is, in a great measure, built up on the imperfect testimony of credulous, hasty, prejudiced, or incompetent witnesses.****

"The enormous polypharmacy of modern times is an excrescence on science, unsupported by any evidence of necessity or fitness, and of which the more complicated formulas are so arbitrary and useless, that, if by any chance they should be forgotten, not one in a hundred of them would ever be re-invented. And as to the chronicles of cure of diseases that are not yet known to be curable, they are written, not in the pages of philosophic observers, but in the tomes of compilers, the aspirations of journalists, and the columns of advertisers.

"It is the part of rational medicine to enlighten the public and the profession in regard to the true powers of the healing art. The community require to be undeceived and re-educated, so far as to know what is true and trustworthy from what is gratuitous, unfounded and fallacious. And the profession themselves will proceed with confidence, self-approval and success, in proportion as they shall have informed mankind on these important subjects. The exaggerated impressions now prevalent in the world, in regard to the powers of medicine, serve only to keep the profession and the public in a false position, to encourage imposture, to augment the number of candidates struggling for employment, to burden and disappoint the community already over-taxed, to lower the standard of professional character, and raise empirics to the level of honest and enlightened physicians." Pp. 55–57.

These liberal extracts will give an idea of the general scope and tendency of the work before us. It should be extensively read by the community at large, and thoroughly studied by every one who pretends to practice medicine in an enlarged and liberal spirit. And if every medical student were henceforth required to study its pages as a pre-requisite to receiving a degree, the Colleges would thereby confer a greater benefit on coming generations than by increasing the number of lectures on therapeutics or enlarging their cabinets of materia medica. In a word, the principles inculcated in this work must hereafter be the guide of every practitioner who, however "regular" he may be in other respects, would not lose all claim to the more distinctive and more honorable title of "Rational Physician." B. E. C.

